# Dose-dense chemotherapy for breast cancer: the story so far

**DOI:** 10.1054/bjoc.2000.1198

**Published:** 2000-05-18

**Authors:** C Hudis

**Affiliations:** Breast Cancer Medicine Service, Memorial Sloan-Kettering Cancer Center, 1275 York Avenue, New York, NY, 10021, USA


British Journal of Cancer (2000) 82(12), 1897–1899
©2000 Cancer Research Campaign
doi: 10.1054/ bjoc.2000.1198, available online at http://www.idealibrary.com on IDEAL®

Editorial

### Dose-dense chemotherapy for breast cancer: the story so far

**C Hudis**

Breast Cancer Medicine Service, Memorial Sloan-Kettering Cancer Center, 1275 York Avenue, New York, NY 10021 USA

This issue of the *British Journal of Cance* contrains two related papers addressing the feasibility of increasing the intensity of adjuvant chemotherapy by increasing the frequency of administration. This approach, called ‘dose-dense’ treatment, is predicated on several related observations and hypotheses. First, fractional cell kill has been observed in selected experimental tumour models, suggesting that while a single cycle of effective cytotoxic chemotherapy may not be curative, multiple cycles should be if the cells are drug-sensitive and the duration is adequate (Skipper, 1971). Secondly, the dose-to-response relationship for some of the most active drugs against breast cancer may not rise continually but instead appears to reach a plateau in the higher end of the normal dose range (Fisher et al, 1997, 1999; Henderson et al, 1998). Thirdly, prior adjuvant treatment with some drugs does not preclude the possibility of a response upon retreatment, suggesting that we are not eliminating all sensitive cells when we use currently available standard agents and regimens (Valagussa et al, 1986). Fourthly, tumour regrowth may be particularly rapid when the number of viable cells is at its lowest because of the Gompertzian shape of the growth curve (Norton et al, 1976). Hence, cell cycle-specific agents might be most useful if applied quickly after a prior treatment when the cells are dividing most rapidly. In addition, this growth pattern is consistent with the observed results of adjuvant therapy trials in terms of their modest impact on outcome and also with the possibility that conventional chemotherapy renders patients much closer to complete cure than it might otherwise seem.

Dose-density is a relative concept. A regimen standing alone cannot really be said to be dose-dense except in comparison to another treatment plan. Treatment with a fixed dose of drug ‘D’ every 2 weeks is therefore more dose-dense than the same drug dose given for the same number of cycles at 3-week intervals. In contrast, ‘dose-intensity’ is a mathematic transformation which takes into account not only dose size but also the relative value of individual drugs and the total duration of treatment. However, it specifically does not account for frequency of administration and assumes, for example, that drug ‘D’ given at dose ‘X’ once every 4 weeks has the same effect as when it is given at 1/4X weekly for four weeks doses. In both cases the dose-intensity calculates as 1/4X per week but the real biological effect could be quite different, particularly if 1/4X is as cytotoxic as X. Indeed, the observations and hypotheses described above predict that even if dose X is four times more effective than 1/4X, it may have a lesser overall impact than the smaller dose given more often. This is the prime motivation behind the testing of dose-dense regimens.

The purest clinical test of dose-dense chemotherapy to date may be the randomized trial of alternating or sequential cyclophosphamide, methotrexate, and 5-fluorouracil (CMF) and doxorubicin (A) (Bonadonna et al, 1995). Over 400 women with four or more involved axillary nodes received four cycles of doxorubicin and eight of CMF. The less dose-dense arm utilized an alternating plan of two cycles of CMF followed by one cycle of doxorubicin (CCACCACCACCA) while the more dose-dense arm consisted of all four cycles of doxorubicin followed by all eight of CMF (AAAACCCCCCCC). At 10 years of follow-up the latter remains significantly superior. To take advantage of the hoped-for steep dose-response relationship for cyclophosphamide, we performed a pilot trial substituting high-dose cyclophosphamide in place of CMF in the sequential plan. Here the dose of cyclophosphamide was escalated and the density of treatment was increased by shortening the inter-treatment interval to 14 days (Hudis et al, 1999*a*). This was made feasible by using granulocyte-colony stimulating Factor. Every 14-day administration of cyclophosphamide had been tested by the Cancer and Leukemia Group B in metastatic disease using granulocyte macrophage-colony stimulating factor, but the use of this regimen within weeks of high-dose doxorubicin had be tested before a randomized trial could be started (Lichtman et al, 1993). Based upon these results, the Southwest Oncology group led a randomized trial (SWOG 9313) of concurrent versus sequential doxorubicin and cyclophosphamide for patients with 0–3 involved lymph nodes, the results of which are awaited.

Based on its promising activity, paclitaxel was then added to the sequential dose-dense regimen of doxorubicin and cyclophosphamide resulting in the ATC regimen comprising three courses of each at 2-week intervals (Hudis et al, 1999*b*). This pilot trial also utilized escalated doses and granulocyte-colony stimulating factor to maintain the planned 2-week dosing intervals so that the nine cycles of treatment were planned to require 18 weeks. Based on the outcome of this and a second small feasibility trial, another SWOG-coordinated randomized trial (SWOG 9623) was begun (Hudis et al, 1996). Here dose-dense sequential doxorubicin, paclitaxel, and cyclophosphamide (ATC) given at 2-week week intervals is being compared to concurrent AC given for four courses at 3-week intervals followed by ‘conventional’ high-dose autologous stem-cell supported consolidation. This trial is open only to patients with 4 or more involved axillary nodes and is about halfway accrued.

Despite these trials, proof that dose-density matters, or at least that it does at the extremes of dosing intervals which require growth factor support, is lacking. The Cancer and Leukemia

*Received 11 January 2000*
*Accepted 23 February 2000*

Group B has already completed a trial in which 2005 patients received four doses of doxorubicin 60 mg $m^{-2}$, cyclophosphamide 600 mg $m^{-2}$, and paclitaxel 175 mg $m^{-2}$ with randomization between every second (more dose-dense) or third (less dose-dense) week treatment intervals. In addition these patients were randomized to treatment with sequential single agents (AAAATTTTCCCC) or concurrent AC followed by paclitaxel. This well-controlled trial will go far in determining the real world value of increasing the dose-density of conventional dose chemotherapy.

The two papers included in this issue add information regarding the feasibility of increasing dose-density. The paper by Bos et al was designed to determine the tolerance for relatively dose-dense CMF administered 2 weeks out of 3 for a total of six cycles as adjuvant treatment (Bos et al, 2000). Because the interval for ‘conventional’ day 1 and 8 CMF has been 4 weeks in most prior regimens, this represents a 25% increase in dose-density. Alternatively, compared to ‘conventional’ intravenous CMF given once every 3 weeks, this represents a 50% increase in dose-intensity and perhaps a doubling of dose-density as well. The use of 750 mg $m^{-2}$ of cyclophosphamide somewhat increases dose-intensity compared to the usual dose of 600 mg $m^{-2}$ used in many other regimens. However, the aforementioned NSABP studies suggest that this slight increase in dose and dose-intensity may not be worthwhile (Fisher et al, 1999; 1997). Nevertheless, among these 23 otherwise-healthy relatively young women (median age 44 years) these investigators clearly demonstrate that a more dose-dense administration of intravenous CMF is feasible if accompanied by granulocyte-colony stimulating factor. They also show that more liberal guidelines for dose-reductions and delays are feasible with CMF, so long as one accepts the increased risks of neutropaenia and anaemia. The most pressing question, however, is whether the patients fare better than with other interventions. For example, it might be that simply adding an anthracycline and/or a taxane, or trastuzumab in appropriate cases, could yield improved results compared to the standard CMF regimen and at lesser cost in terms of toxicity (EBCTCG, 1998; Henderson et al, 1998) At the same time, it is worth noting that for a variety of reasons, a significant proportion of patients is routinely treated with CMF and a better CMF could therefore be important.

Given the apparent superiority of anthracycline-containing regimens seen in the overview, the global trend towards increased use among many subsets of patients, and the demonstration that paclitaxel adds to standard adjuvant AC and increases the activity of single-agent doxorubicin, the second paper, by Lalisang et al, is particularly timely (EBCTCG, 1998; Lalisang et al, 2000). Their testing of the ET combination is rational given that AT was very active in phase II studies and epirubicin may offer decreased cardiotoxicity with no loss of efficacy (Dombernowsky et al, 1995; Gianni et al, 1995; Jain et al, 1985). Here they carefully determined the feasibility of using granulocyte-colony stimulating factor to reduce the interval between doses of ET. Thus, this study tested the feasibility of increasing dose-density while maintaining dose-level.

Subsequently, after establishing the ‘MTD’ for ET (75/135 mg $m^{-2}$) as a 10-day dosing interval, they tested 75/175 and showed this to be feasible at 10-day intervals as well. At this interval, they judged the toxicity to be mild and, compared to recent studies of high-dose autologous stem cell-supported programs, they are correct. However, again toxicity was certainly more than one would anticipate with AT or ET given at 21-day intervals, and the worth of the shorter more dense treatment plan must be established before considering this for general use. In addition to the randomized studies described above, there are several adjuvant therapy trials planned or underway which seek to determine the benefits of more dose-intensive and dose-dense treatment plans utilizing EC and ET in the adjuvant setting and these must be completed before we can begin to judge the worth of more dose-dense ET.

Do these two studies advance our field? The answer to this question is maybe, but only moderately. For sure these studies add to the literature showing that more dose-dense regimens are feasible largely through the use of granulocyte-colony stimulating factor. At least one study from the pre-growth factor era suggests that dose-density matters, but to date no study has established that this last increment of dose-density – the use of standard doses at sub-3 week intervals – made feasible through the use of growth factors, is worthwhile. Given the disappointing and somewhat surprising results of the three large North American trials of dose and dose-intensity for doxorubicin (Cancer and Leukemia Group B 9344) (Henderson et al, 1998) and cyclophosphamide (Fisher et al, 1997; 1999) we should not take for granted that increased dose-density or dose-intensity will improve outcomes. We can hope that this is so and we can hypothesize that this is so but we owe it to our patients to quickly and efficiently address the value of dose-density through appropriate randomized studies. Until then, these approaches remain exciting but not quite ready for routine use.

### REFERENCES

Bonadonna G, Zambette M and Valagussa P (1995) Sequential or alternating doxorubicin and CMF regimens in breast cancer with more than three positive nodes. *JAMA* **273**: 542–547

Bos A, de Graf H, de Vries E, Piersma H and Willemse P (2000) Feasibility of a dose-intensive CMF regimen with granulocyte colony stimulating factor as adjuvant therapy in premenopausal patients with node-positive breast cancer. *Br J Cancer* **82**: 1920–1924

Dombernowsky P, Gehl J, Boesgaard M, Jensen T, Jensen B and Ejlersen B (1995) Treatment of metastatic breast cancer with paclitaxel and doxorubicin. *Semin Oncol* **22**: 13–17

EBCTCG (1998) Polychemotherapy for early breast cancer: an overview of the randomised trials. *Lancet* **352**: 930–942

Fisher B, Anderson S, DeCillis A, Dimitrov N, Atkins J, Fehrenbacher L, Henry P, Romond E, Lanier K, Davila E, Kardinal C, Laufman L, Pierce H, Abramson N, Keller A, Hamm J, Wickerham D, Begovic M, Tan-Chiu E, Tian W and Wolmark N (1999) Further evaluation of intensified and increased total dose of cyclophosphamide for the treatment of primary breast cancer: findings from the National Surgical Adjuvant Breast and Bowel Project B-25. *J Clin Oncol* **17**: 3374–3388

Fisher B, Anderson S, Wickerham D, DeCillis A, Dimitrov N, Mamounas E, Wolmark N, Pugh R, Atkins J, Meyers F, Abramson N, Wolter J, Bornstein R, Levy L, Romond E, Caggiano V, Grimaldi M, Jochimsen P and Deckers P (1997) Increased intensification and total dose of cyclophosphamide in a doxorubicin-dyclophosphamide regimen for the treatment of primary breast cancer: findings from National Surgical Adjuvant Breast and Bowel Project B-22. *J Clin Oncol* **15**: 1858–1869

Gianni L, Munzone E, Capri G, Fulfaro F, Tarenzi E, Villani F, Spreafico C, Laffranchi A, Caraceni A, Martini C, Stefanelli M, Valagussa P and Bonadonna G (1995) Paclitaxel by 3-hour infusion in combination with bolus doxorubicin in women with untreated metastatic breast cancer: High antitumor efficacy and cardiac effects in a dose finding and sequence-finding study. *J Clin Oncol* **13**: 2688–2699

Henderson I, Berry D, Demetri G, Cirrincione C, Goldstein L, Martino S, Ingle J, Cooper M, Canellos G, Borden E, Fleming G, Holland J, Graziano S, Carpenter J, Muss H and Norton L (1998) Improved disease-free and overall survival from the addition of sequential paclitaxel but not from the escalation of doxorubicin dose level in the adjuvant chemotherapy of patients with node-positive primary breast cancer. *Proceedings of the Annual Meeting of the American Society of Clinical Oncology* **17**: abstract 390a

 *© 2000 Cancer Research Campaign*

Hudis C, Seidman A, Raptis G, Baselga J, Fennelly D, Theodoulou M, Gilewski T, Currie V, Moynahan M, Surbone A, Sklarin N, Robles M, Riccio L, Yao, T.-J, Uhlenhopp M and Norton L (1996) Sequential dose-dense doxorubicin –> paclitaxel –> cyclophosphamide is less toxic than doxorubicin –> concurrent paclitaxel + cyclophosphamide as adjuvant therapy in resected node positive breast cancer. *Proc Am Soc Clin Onc* **15**: 119

Hudis C, Fornier M, Riccio L, Lebwohl D, Crown J, Gilewski T, Surbone A, Currie V, Seidman A, Reichman B, Moynahan M, Raptis G, Sklarin N, Theodoulou M, Weiselberg L, Salvaggio R, Panageas K, Yao T and Norton L (1999*a*) Five-year results of dose-intensive sequential adjuvant chemotherapy for women with high-risk node-positive breast cancer: a phase II study. *J Clin Oncol* **17**: 1118–1126

Hudis C, Seidman A, Baselga J, Raptis G, Lebwohl D, Gilewski T, Moynahan M, Sklarin N, Fennelly D, Crown J, Surbone A, Uhlenhopp M, Riedel E, Yao T and Norton L (1999*b*) Sequential dose-dense doxorubicin, paclitaxel, and cyclophosphamide for resectable high-risk breast cancer: feasibility and efficacy. *J Clin Oncol* **17**: 93–100

Jain KK, Casper ES, Geller NL and Al E (1985) A prospective randomized comparison of epirubicin and doxorubicin in patients with advanced breast cancer. *J Clin Oncol* **3**: 3818–3826

Lalisang R, Voest E, Wils J, Nortier H, Erdkamp F, Hillen H, Wals J, Schouten H and Blijham G (2000) Dose-dense epirubicin and paclitaxel with G-CSF: a study of decreasing intervals in metastatic breast cancer. *Br J Cancer* **82**: 1914–1919

Lichtman S, Ratain M, Van Echo D, Egorin M, Budman D, Vogelzang N, Norton L, Rosner G and Schilsky R (1993) Phase I trial of granulocyte-macrophage colony stimulating factor (GM-CSF) plus high dose biweekly cyclophosphamide: a CALGB Study. *J Natl Cancer Inst* **85**: 1319–1326

Norton L, Simon R, Brereton J and Bogden A (1976) Predicting the course of Gompertzian growth. *Nature* **264**: 542–545

Skipper HE (1971) Kinetics of mammary tumor cell growth and implications for therapy. *Cancer* **28**: 1479–1499

Valagussa P, Tancini G and Bonadonna G (1986) Salvage treatment of patients suffering relapse after adjuvant CMF chemotherapy. *Cancer* **58**: 1411

© 2000 Cancer Research Campaign

